# How to deal with a clustering effect in the assessment of a hand hygiene improvement strategy implemented worldwide?

**DOI:** 10.1186/1753-6561-5-S6-O71

**Published:** 2011-06-29

**Authors:** A Gayet-Ageron, B Allegranzi, H Attar, D Pittet

**Affiliations:** 1 Infection Control Programme, Universiy Hospitals of Geneva, Geneva, Switzerland; 2 World Health Organization, Geneva, Switzerland

## Introduction / objectives

Hand hygiene (HH) is a key measure to prevent healthcare-associated infections. Between 2006 and 2008, the WHO conducted pilot testing of the implementation of a multimodal HH improvement strategy in six sites worldwide. Collected data presented several aspects of complexity and levels of clustering that needed to be taken into account in the analysis. We describe a statistical approach aimed at minimizing potential bias arising from such complex datasets.

## Methods

Through a before/after observational study, HH compliance was assessed in several wards ofdifferent hospitals in five countries from April 2006 to December 2008 using a validated method. The HH opportunity was the unit of analysis; data on HH indications and actions, observed professional categories, and day of the week observed were available. Simple logistic regression and mixed models using a nested clustering effect (hospital\ward\session) were used to assess the effect of the WHO strategy.

## Results

A total of 45 420 HH opportunities were collected during 3613 sessions in 94 wards inin 43 hospitals from six pilot sites. Using a logistic regression model with fixed effects only, the crude odds for HH compliance after the intervention were 1.71 (95% CI: 1.65-1.78) and the adjusted odds, 1.80 (95% CI: 1.73-1.88). With a mixed model, the crude odds for HH compliance after intervention were 1.99 (95% CI: 1.86-2.14) and 2.13 (95% CI: 1.97-2.29) after adjustment.

## Conclusion

The effect of the strategy implementation may be underestimated if the correlation existing between data is not taken into account. A generalized linear mixed model using a nested clustering effect may be a solution to deal with correlated data, but as paired data are lacking, all correlations cannot be taken into account.

## Disclosure of interest

None declared.

